# Age-Corrected Beta Cell Mass Following Onset of Type 1 Diabetes Mellitus Correlates with Plasma C-Peptide in Humans

**DOI:** 10.1371/journal.pone.0026873

**Published:** 2011-11-02

**Authors:** David J. Klinke

**Affiliations:** 1 Department of Chemical Engineering, West Virginia University, Morgantown, West Virginia, United States of America; 2 Department of Immunology, Microbiology, and Cell Biology, West Virginia University, Morgantown, West Virginia, United States of America; University of Las Palmas de Gran Canaria, Spain

## Abstract

**Background:**

The inability to produce insulin endogenously precipitates the clinical symptoms of type 1 diabetes mellitus. However, the dynamic trajectory of beta cell destruction following onset remains unclear. Using model-based inference, the severity of beta cell destruction at onset decreases with age where, on average, a 40% reduction in beta cell mass was sufficient to precipitate clinical symptoms at 20 years of age. While plasma C-peptide provides a surrogate measure of endogenous insulin production post-onset, it is unclear as to whether plasma C-peptide represents changes in beta cell mass or beta cell function. The objective of this paper was to determine the relationship between beta cell mass and endogenous insulin production post-onset.

**Methods and Findings:**

Model-based inference was used to compare direct measures of beta cell mass in 102 patients against contemporary measures of plasma C-peptide obtained from three studies that collectively followed 834 patients post-onset of clinical symptoms. An empirical Bayesian approach was used to establish the level of confidence associated with the model prediction. Age-corrected estimates of beta cell mass that were inferred from a series of landmark pancreatic autopsy studies significantly correlate (*p*>0.9995) with contemporary measures of plasma C-peptide levels following onset.

**Conclusions:**

Given the correlation between beta cell mass and plasma C-peptide following onset, plasma C-peptide may provide a surrogate measure of beta cell mass in humans. The clinical relevance of this study is that therapeutic strategies that provide an increase in plasma C-peptide over the predicted value for an individual may actually improve beta cell mass. The model predictions may establish a standard historical “control” group - a prior in a Bayesian context - for clinical trials.

## Introduction

The regulation of human metabolism is a complicated process that has evolved to match the intermittent nature of the availability of metabolic substrates with the constant energetic requirements for life [1]. The dysregulation of this process may manifest itself in multiple ways, including clinical presentation with the symptoms of diabetes. The societal burden of diabetes is significant through increased health care costs and reduced human productivity [2]–[4]. Conservative estimates predict that the number of people with diabetes will more than double between 2000 and 2030 [5]. Reducing the overall impact of this disease requires an improved understanding of the aetiology of diabetes.

Understanding the aetiology of type 1 diabetes mellitus is challenging due to the inability to observe directly the events in the human pancreas that lead to the onset of hyperglycemia [6]. While a reduction in endogenous insulin production precipitates the onset of hyperglycemia, it is commonly stated that the onset of hyperglycemia occurs when 80–95% of an individual's beta cells are destroyed [7], [8]. However, this common wisdom is based largely on a small number of biopsy studies from individuals with recent disease onset who died soon after diabetes onset (e.g., [9]–[11]). One might infer from this common wisdom that the ability to enhance beta cell function or preserve the remaining beta cells would have a limited therapeutic potential [12]. As a result, the research effort has focused on developing prognostic tools for identifying individual, who will develop type 1 diabetes, prior to onset. Given the clinical importance of this question, the objective of a recent study [13] was to test the common wisdom for the pathophysiology of type 1 diabetes mellitus against the histopathological evidence.

A meta-analysis was used to extract and assess the significance of embedded trends within these landmark studies. The data reported in these landmark studies provide measurements of the remaining beta cells (i.e. beta cell mass) at the time of death. Patients included in these studies died between 0 and 117 months following diagnosis. While beta cell mass or endogenous insulin production are not measured directly following onset, plasma C-peptide is used as a surrogate measure of endogenous insulin production [16]–[18]. The measurement of C-peptide in a cohort of patients with type 1 diabetes has been shown to vary non-linearly with time following onset. These measurements suggest that endogenous insulin production increases following onset of type 1 diabetes and then slowly declines in the subsequent years. Therefore, inferring the beta cell mass at onset must control for this variability in the time of beta cell mass measurement. By limiting the analysis to a subset of patients who died within three weeks following diagnosis, the percent reduction in beta cell mass at onset is not fixed but varies with age [13]. This trend suggests that, in a 20-year old individual, as little as a 40% reduction in beta cell mass is sufficient to precipitate clinical symptoms of type 1 diabetes. As this trend is at odds with the existing model for the natural history of the disease [19], a mathematical model, which was created based upon physiological considerations, explains this behavior [13]. Here, this physiology-based mathematical model was used to predict the change - relative to age-corrected non-diabetic controls - in beta cell mass post-onset in the entire cohort of patients reported in these landmark studies. While changes in plasma C-peptide may be due to either a reduction in beta cell function or a reduction in beta cell mass, the recovery in plasma C-peptide following onset is interpreted commonly as a recovery in beta cell function due to therapy. To test this common interpretation, the model predictions were used to assess whether, following onset, the dynamics of beta cell mass correlate with the dynamics of plasma C-peptide measurements obtained in a cohort of more contemporary patients.

## Results and Discussion

**Figure 1:**
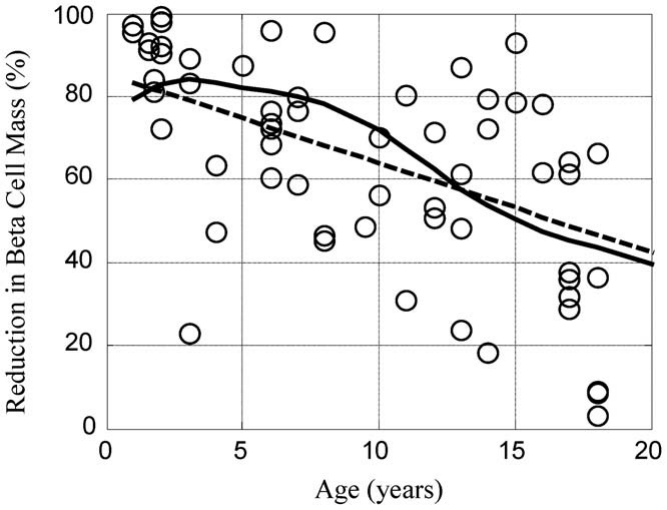
The growth rates for beta cell mass and total body weight exhibit different dynamic trends based upon age. The age-associated changes in total beta cell mass (dotted line) corresponds to the product of the dynamic trends in beta cell density [11] and volume of the pancreas [11]. The change in body weight (solid line) as a function of age is an average value from male and female growth charts [20]. A similar figure appears in [13] but is included here for continuity.

Growth of the human body is a dynamic non-linear process where different parts of the body grow at different rates. Of particular relevance to type 1 diabetes mellitus, body weight changes [20] at a different rate than beta cell mass [11], as shown in Figure 1. One possible explanation for the observed reduction in beta cell mass at onset could be attributed to the dynamic imbalance between the number of beta cells and the insulin requirements for a growing body. A mathematical model was created to test whether this explanation provides a better representation of the data.

The rate of change in insulin in the body can be represented as a dynamic balance between the source of insulin, which is proportional to beta cell mass, and sinks for insulin, which are proportional to body weight:
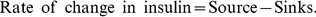
(1)


This relationship can be expressed in terms of a differential equation:
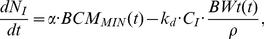
(2)


where 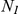 is the number of insulin molecules in the body; 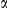 is the maximum rate of insulin release per beta cell mass (BCM), in units of 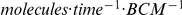; 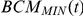 is the minimum required beta cell mass to maintain euglycemia; 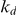 is the rate constant for insulin clearance from the body, in units of 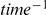; 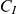 is the plasma concentration of insulin, in units of 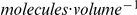; 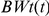 is the body weight, in units of 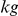, that changes with age, 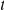 (see Figure 1); and 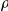 is the average density of the human body, in units of 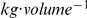. Under fasting conditions, the source and sinks are exactly balanced such that the rate of change of insulin is equal to zero (i.e., 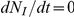). Under fasting conditions, terms in equation 2 can be rearranged to solve for 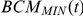:
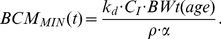
(3)


By defining 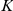 to be equal to 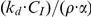, equation 3 simplifies to
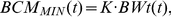
(4)


which implies that the minimum required beta cell mass is proportional to dynamic changes in body weight, shown by the solid curve in Figure 1.

The total beta cell mass (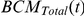) was represented as the product of the beta cell density in the pancreas times the total weight (volume) of the pancreas, shown as a dotted line in Figure 1. This provides an estimate of the change in beta cell mass as a function of age in normal individuals. In addition, the total beta cell mass can be represented as the sum of the minimum beta cell mass (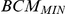) and excess, or reserve capacity, beta cell mass (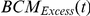):
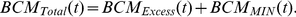
(5)


Equation 5 can be rearranged, combined with equation 4, and divided by the total beta cell mass to define the normalized excess beta cell mass (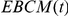):
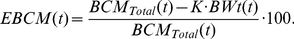
(6)


**Figure 2:**
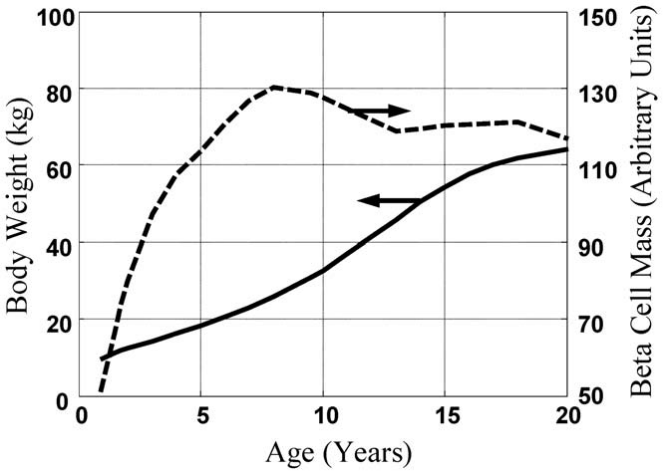
Comparison between the predicted and measured excess beta cell mass in recent onset patients. The measured reduction in beta cell mass in 60 patients, under 20 years of age, that died within three weeks of diagnosis of type 1 diabetes mellitus (circles [13]) is compared against the excess beta cell mass predicted by the physiology-based mathematical model (Equation 6 - solid curve) and a trendline obtained by linear regression (dotted line). A similar figure appears in [13] but is included here for continuity.

This mathematical model was used to predict the “excess” beta cell mass (EBCM) as a function of age 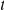 by capturing the dynamic balance between changes in body weight and beta cell mass. The “excess” beta cell mass corresponds to the reduction in beta cell mass that is required before hyperglycemia occurs. EBCM corresponds to the ratio of insulin-deficient islets to the total number of islets observed in the transected pancreas reported in recent onset patients [9]–[11], as the size of the pancreas in recent onset patients was unchanged relative to normal controls [13]. The resulting model prediction for EBCM as a function of age is shown in Figure 2 (solid line). The observed reduction in beta cell mass in pancreata obtained from the subset of recent onset patients (i.e., died within three weeks of diagnosis) are shown for comparison. The parameter 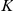 was estimated to be 499 units of BCM 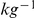 (95% C.I. = 458 to 605) [13]. The EBCM relationship exhibits a similar dependence with age, as the youngest patients exhibited an 85% reduction in beta cell mass while only a 40% reduction was observed by the age of 20. In other words, the beta cell mass initially grows at a faster rate relative to the whole body. The beta cell mass plateaus while the overall body weight steadily increases through 20 years of age. The net result of the different growth dynamics is that the “excess” beta cell mass declines with age. In addition, the mathematical model provides a prediction of the beta cell mass required to maintain glucose homeostasis as a function of the patient's age 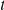. In the following paragraphs, we will use these predictions of the minimum required beta cell mass to interpret plasma C-peptide levels following onset.

### Comparison of beta cell mass to plasma C-peptide levels

The steady state (i.e., fasting) concentration of insulin in the body can be obtained by rearranging Equation 3:
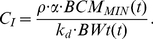
(7)


**Figure 3:**
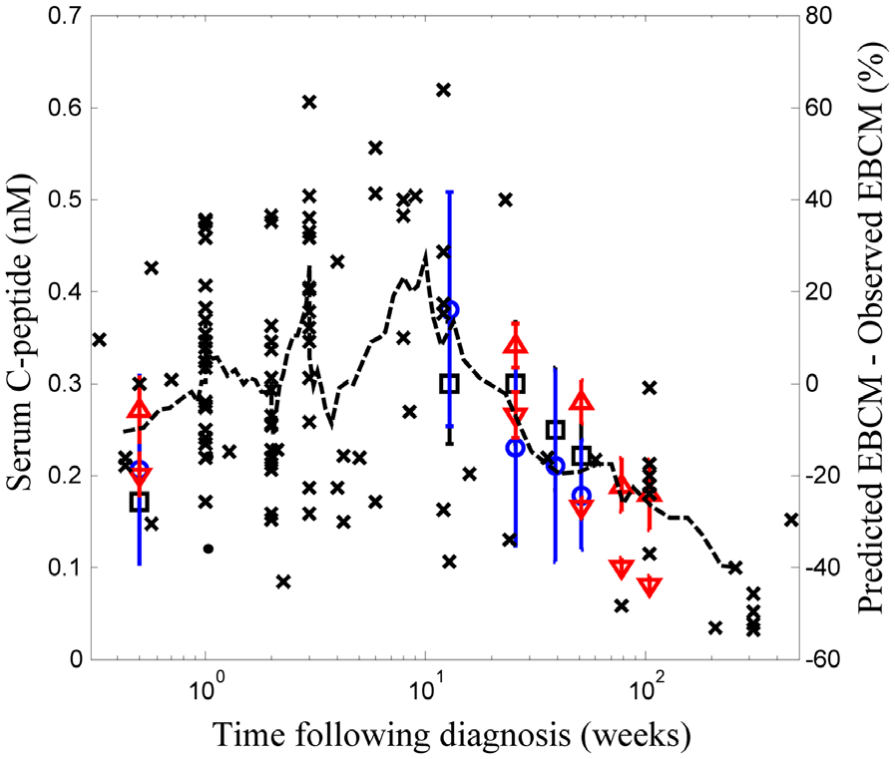
Dynamic change in residual beta cell mass corresponds to the dynamic change in plasma C-peptide following onset of type 1 diabetes. The difference between predicted and observed excess beta cell mass (right axis: x) and plasma C-peptide (left axis: square [17], circle [18], ▵- children initially negative for autoantibodies at diagnosis and during follow-up [16], and ▿- children positive for at least one autoantibody [16]) shown as a function of time following clinical diagnosis of type 1 diabetes. Plasma C-peptide levels are reported as a mean + SE. A 9-point moving average of the difference in excess beta cell mass is shown for comparison (dotted line). The dynamic change in observed beta cell mass was obtained from pancreata obtained from patients with type 1 diabetes [9]–[11]. The predicted beta cell mass is an estimate of the minimum beta cell mass required to maintain glucose homeostasis.

This relationship implies that steady state levels of insulin can be maintained when the 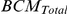 is greater than 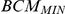 by reducing the insulin production per cell (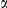). Conversely, 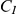 will decrease below fasting levels when the 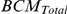 is less than 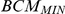, as an increase in 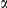 can not be used to compensate for a reduction in beta cell mass. To compare our model against the plasma C-peptide data, we implicitly make a couple of assumptions. First, an equivalent expression for Eqn 7 can be developed for plasma C-peptide such that the observed level of plasma C-peptide is proportional to the predicted concentration of insulin (i.e., 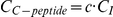, where 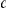 is a proportionality constant that does not change with disease state). Second, 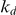 for plasma C-peptide does not depend on C-peptide concentration or disease state. In addition, the model was originally developed using data limited to recent onset patients (i.e., died within three weeks of diagnosis). This was done so that the predicted value for EBCM should match the observed changes in insulin positive islets, i.e., predicted EBCM minus observed EBCM should equal zero. When considering the entire cohort, the age of the individual was used to predict a EBCM using the corresponding values for 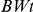 and 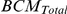. The comparison between plasma C-peptide levels and the predicted EBCM minus observed EBCM are both shown as a function of time following diagnosis in Figure 3.

A predicted EBCM greater than the observed EBCM suggests that there are less insulin deficient islets than would be expected to maintain euglycemia given the age of the individual (i.e., 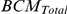 is greater than 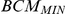) and suggests a recovery of beta cell mass following onset. A predicted EBCM less than the observed EBCM suggests that there is a further decline in beta cell mass (i.e., 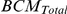 is less than 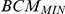). The trend in the post-mortem studies suggests a slight rise in the 10 weeks following diagnosis followed by a progressive decline in beta cell mass. The observed plasma C-peptide levels show a similar dynamic trend.

**Figure 4:**
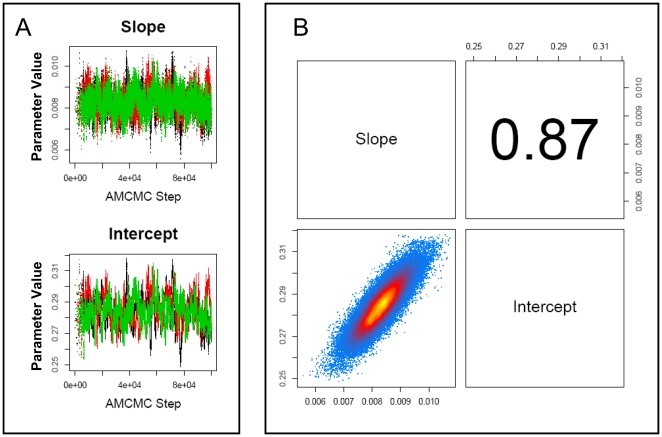
Markov Chain Monte Carlo summary plots for the model parameters. (A) The trace of slope and intercept parameters is shown as a function of MCMC step. The traces for three parallel chains are shown in different colors: Chain 1 (Green), Chain 2 (Black), and Chain 3 (Red) (B) Projection of the marginalized posterior probability density for the slope and intercept given the available data. Each point in the scatter plot represents an individual step obtained from three parallel Markov Chains each containing 100,000 MCMC steps. The density of points is represented by the color (yellow - highest density; blue - lowest density) and estimated using kernel density estimation. The correlation coefficient of the parameters derived from all three Markov chains is shown above the diagonal.

**Figure 5:**
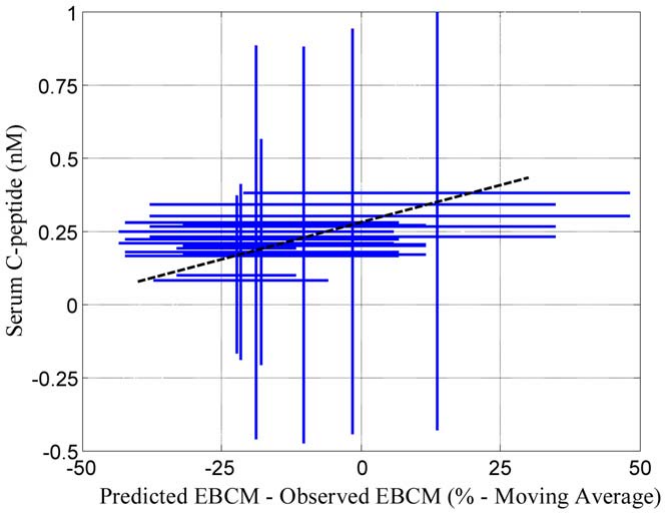
Correlation between residual beta cell mass and plasma C-peptide following onset of type 1 diabetes. Reported plasma C-peptide values (y-values) are shown against the moving average value, shown in Figure 3, for the difference between predicted and observed excess beta cell mass (x-values) at the same time. The dotted line highlights the linear trend (*p*>0.9995 that the slope of the line is greater than zero). The standard deviations in plasma C-peptide and excess beta cell mass are represented by size of the crosses.

To establish whether changes in plasma C-peptide levels and excess beta cell mass exhibit a statistically significant correlation, values for plasma C-peptide levels and the predicted minus observed excess beta cell mass obtained at the same time following diagnosis were plotted against each other. Linear regression was used to establish whether these two estimates of beta cell function exhibit a positive correlation. An empirical Bayesian approach was used to establish the statistical significance of the correlation (i.e., the slope is greater than 0 with greater than 95% confidence) [22]. A summary of the Markov Chain Monte Carlo results are shown in Figure 4. These results indicate that the three Markov Chains: 1) are independent, 2) randomly sample the same region of parameter space, 3) provide estimates of the slope and intercept that are correlated and can not be uniquely determined, and 4) predict that the slope is greater than zero despite the correlation. The positive correlation between plasma C-peptide and the difference between predicted and observed EBCM is shown in Figure 5. As illustrated by the posterior distribution in the slope parameter (see Figure 4B), the probability that these two quantities exhibit a positive correlation (i.e., the slope of the dotted line is greater than zero) is greater than 99.95%. These results also suggest that there is a regrowth of beta cell mass following the onset of clinical symptoms.

The use of models to aid in understanding system behavior is a central theme in science that transcends disciplinary boundaries [23]. In this meta-analysis of the extent of beta cell destruction in patients with type 1 diabetes, two competing theories were represented as mathematical models. The first model corresponds to the prevailing theory that the degree of beta cell reduction at onset is a fixed value. The second model, the physiological model, corresponds to the idea that the observed reduction is a result of a dynamic balance between beta cell mass and body weight. Previously we show that the physiological model exhibits better predictive accuracy, given the available data and the similar complexity between the models, as each model contains a single adjustable parameter [13]. In this work, the simplified physiological model was used to show that age-corrected changes in beta cell mass correlated with changes in endogenous insulin production following diagnosis, as an external validation of the model [21]. A correlation between endogenous insulin production and beta cell mass has been previously shown in animal models [24], [25] and in humans following pancreatectomy [26], [27] and islet transplantation [28], [29]. To my knowledge, this is the first analysis of data obtained during the natural history of type 1 diabetes. Given the correlation between age-corrected beta cell mass and plasma C-peptide following onset, plasma C-peptide may provide a surrogate measure of beta cell mass in humans that is adjusted for age. This work also suggests that therapeutic strategies that provide an increase in plasma C-peptide greater than the observed range in values for a given time following diagnosis may actually improve beta cell mass. Longitudinal studies that measure beta cell mass and body size in patients may be used in the future to validate these predictions.

In summary, this physiological model suggests that clinical presentation of the disease is not attributed solely to the destruction of beta cell mass but is the result of a dynamic imbalance between the production of insulin (i.e., beta cell mass) and the size of the system (i.e., body weight). The correlation between the model predictions and the reported changes in plasma C-peptide suggests two points. First, the methods that were used in these landmark studies exhibit a certain degree of accuracy in estimating beta cell mass. By considering the trends in the data, we are able to correct for the imprecision of the assays used. This implies a subtle but important point. Inference of knowledge from data is limited by the signal-to-noise ratio of the assay used to measure a biological state (e.g., [30]). Common metrics used to assess how well an interpretation (i.e., a model) describes data, such as a coefficient of correlation (i.e., 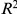), lump the reproducibility of an assay (i.e., the noise) together with model inadequacy (i.e., the difference between the model and the underlying biological signal). Conclusions drawn from studies that evaluate the predictive potential of different biological metrics and that do not distinguish between the underlying sources of variation are fundamentally flawed (e.g., [27]). By using an empirical Bayesian approach, we are able establish a level of confidence in the biological interpretation of the data that is independent of the underlying noise in the system. Second, the similar dynamic trends suggest that the natural history of the disease is similar across these studies. In part, this similarity reflects the difficulty in changing the natural history therapeutically [14]. This dynamic trajectory in plasma C-peptide may provide a standard historical “control” group for non-placebo-controlled trials, as suggested in a recent editorial [15]. From a Bayesian perspective, this dynamic trajectory, expressed in the form of a mathematical model, provides prior knowledge - inferred from the clinical data obtained from 936 patients spread across multiple continents and 40+ years - for interpreting new data. The distribution in these trajectories provides a context for establishing a level of belief in whether a therapeutic agent provides a significant clinical benefit. In silico model-based inference may be particularly helpful for screening multiple promising candidates in small non-placebo-controlled trials. Therapeutic candidates that demonstrate a significant clinical benefit, given the prior, may then be tested in larger double-blind placebo-controlled trials, the current gold standard for FDA approval. Such a staged strategy may reduce the overall cost and time associated with approval and, ultimately, improve clinical outcomes.

## Methods

In silico model-based inference was used to establish whether changes in beta cell mass correlated with changes in endogenous insulin production in individuals diagnosed with type 1 diabetes. Beta cell mass was estimated from three landmark histopathology studies that report insulin positive cells in pancreatic biopsies from patients exhibiting symptoms of Type 1 diabetes mellitus [9]–[11]. Endogenous insulin production was estimated from plasma C-peptide levels measured in patients with type 1 diabetes [16]–[18]. An empirical Bayesian approach [22] was used to establish a level of confidence associated with the correlation between beta cell mass and plasma C-peptide levels. The age-associated changes in total beta cell mass (dotted line) was interpolated using the product of two cubic splines that were fit to the dynamic trend in beta cell density [11] and to the age-associated change in volume of the pancreas [11]. The change in body weight (solid line) as a function of age was interpolated using a cubic spline that was fit to the average value from male and female growth charts [20]. Additional details regarding how the data was analyzed are described in the following paragraphs.

### Islet Histopathology Study Selection

Changes in beta cell mass in patients with type 1 diabetes were obtained from a series of histopathology studies, which reported insulin positive cells in pancreatic biopsies from patients exhibiting symptoms of Type 1 diabetes mellitus. Three studies were identified where histopathologies of the endocrine pancreas were quantitatively reported for a group of young patients. All of the patients were under the age of 25 and had died from primarily diabetic ketoacidosis [9]–[11]. In total, 102 unique histopathology results were included from these three studies.

### C-peptide Study Selection

C-peptide is a protein that assists in the synthesis of insulin by the beta cells and is released in the secretory granules in a 1∶1 molar ratio with insulin. In the presence of exogenous insulin, plasma C-peptide is used as a surrogate measure of endogenous insulin production following onset of type 1 diabetes. Three studies were identified where longitudinal studies were used to report the plasma C-peptide levels as a function of time following diagnosis for type 1 diabetes [16]–[18]. Two studies report fasting plasma C-peptide levels [17], [18] while the third reports random measurements [16]. In addition, one study stratified the reported plasma C-peptide levels into two groups: individuals that tested positive for at least one islet cell specific autoantibody (e.g., islet cell antibodies (ICA), insulin autoantibodies (IAA), and glutamic acid decarboxylase (GAD65A)) and individuals that were negative for autoantibody expression [16]. The presence of islet cell specific autoantibodies was a requirement for inclusion in Chaillous et al., while in Pozzilli et al. all of the results were reported together irrespective of autoantibody status. The results are reported as a mean 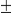 standard error. In total, the results for 834 patients were included from these three studies, as summarized in Table 1.

**Table 1:** Summary statistics for the studies that report plasma C-peptide levels.

Study	Age	Study	N	Autoantibody
	Range	Duration		Status
Komulainen et al. [16]	0.8–14.9 yrs	24 months	769	732 AA+/37 AA-
Pozzilli et al. [17]	<15 yrs	12 months	24	–
Chaillous et al. [18]	7–40 yrs	12 months	41	all AA+

### Statistical Analysis of Age-Corrected Reduction in Beta Cell Mass Compared to Plasma C-peptide Levels

We hypothesized that a decrease or an increase in beta cell mass relative to the expected beta cell mass for the age of an individual should correspond to changes in plasma C-peptide levels. The plasma C-peptide levels are reported as aggregate values at a given time point following diagnosis. In contrast, observations of beta cell mass are reported for single individuals at their corresponding time of death following diagnosis. To establish a fair comparison, an average value for the predicted minus observed excess beta cell mass was estimated using a nine-point moving average and centered at the time following diagnosis (dotted line in Figure 3). The uncertainty in the average value was also estimated using a nine-point moving standard deviation.

An empirical Bayesian approach was used to establish the level of confidence associated with the correlation coefficient between these two estimates of beta cell function (i.e., the linear regression slope), given the available data [22]. The data was weighted based upon the sample size. A Markov chain Monte Carlo algorithm was used to estimate the posterior distribution in the slope and intercept of the linear regression line. An initial unbiased Gaussian prior distribution was used to propose new steps in the Markov chain. The prior distribution was scaled to achieve an acceptance fraction of 0.4. The Gelman-Rubin potential scale reduction factor was used to estimate convergence of three independent Markov chains to the posterior distribution [31]. Posterior estimates of the slope were obtained from the converged segments of the three independent chains. Each of the chains contained greater than 100,000 steps following convergence. A p-value of greater than 0.95 was considered significant (i.e., greater than 95% of the steps in the converged Markov chains exhibited a slope greater than 0).
